# Anxiety and Depression Mediate the Health-Related Quality of Life Differently in Patients with Cardiovascular Disease and Stroke–Preliminary Report of the Yilan Study: A Population-Based Community Health Survey

**DOI:** 10.1371/journal.pone.0107609

**Published:** 2014-09-16

**Authors:** Nai-Wei Hsu, Hsuan-Ming Tsao, Hsi-Chung Chen, Pesus Chou

**Affiliations:** 1 Division of Cardiology, Department of Internal Medicine, National Yang-Ming University Hospital, Yilan, Taiwan; 2 Department of Medicine, School of Medicine, National Yang-Ming University, Taipei, Taiwan; 3 Department of Psychiatry and Center of Sleep Disorders, National Taiwan University Hospital, Taipei, Taiwan; 4 Community Medicine Research Center & Institute of Public Health, National Yang-Ming University, Taipei, Taiwan; Xi'an Jiaotong University School of Medicine, China

## Abstract

**Background:**

Cardiovascular disease and stroke have emerged as substantial and growing health challenges to populations around the world. Besides for the survival and medical prognosis, how to improve the health-related quality of life (HRQoL) might also become one of the goals of treatment programs. There are multiple factors that influence HRQol, including comorbidity, mental function and lifestyle. However, substantial research and investigation have still not clarified these underlying pathways, which merit further attention. The purpose of this study was to determine how psychological factors affect the link between cardiovascular disease and stroke with HRQoL.

**Methods and Result:**

A total of 1,285 elder subjects at least 65 years of age (47.2% male) were enrolled. The mental function and HRQol of each patient was then measured using the Hospital Anxiety and Depression Scale and Short Form-12. After multiple regression analysis, anxiety, depression, cardiovascular disease, stroke, education level and age were shown to be associated with both mental component score (MCS) and physical component score (PCS). In the mediation analysis using the SPSS macro provided by Preacher and Hayes, cardiovascular disease and stroke affected HRQoL via anxiety and depression, respectively.

**Conclusions:**

These results suggest that cardiovascular disease and stroke have negative impacts on patient MCS and PCS through different underlying pathways. Cardiovascular disease influences the HRQoL both directly and indirectly with the mediation of anxiety, and stroke influences the HRQoL by way of depression. These findings support the proposition that different combinations of both physical and psychological support are necessary to best manage these diseases.

## Introduction

Cardiovascular disease and stroke have become potent and emerging threats to the world’s health care systems. In 2008, cardiovascular disease and stroke caused more than 17 million deaths around the globe. [1] Furthermore, it has been projected that these diseases will become the two leading causes of death from 2020 to 2030. [2], [3] However, most patients with cardiovascular disease and stroke can at best only be well-controlled and not cured. Therefore, many experts have suggested that maintaining and even improving the quality of life (QoL) for those patients, especially the elderly, might be one of the major goals of treatment [4]–[6].

Quality of life has been reported to be influenced by many factors with rather complex interactions. [5], [7] Therefore, a conceptual model might be beneficial in explaining the relationships among such factors. [5] Wilson and Cleary have already suggested a 5-level model, including biological, physical and mental symptoms, functions, health satisfaction, age and environmental conditions, trying to classify and clarify the relationships between these variables. [8] By this model, Halvorsrud et al. demonstrated that QoL can be predicted by factors such as health satisfaction directly, and by environmental conditions through health satisfaction and depressive symptoms indirectly. [5] Therefore, health-related quality of life (HRQoL) seems to play an important role in influencing the QoL in people with cardiovascular disease and stroke.

Health-related quality of life in patients with cardiovascular disease, stroke, and other chronic diseases has been studied extensively using different methods in recent years. [6], [7], [9]–[19] The results of these studies have suggested that the HRQoL might be associated with lifestyle, comorbidity and mental health. [6], [7], [9], [10], [12]–[15], [17] Some studies disclosed that although chronic diseases usually exert a negative impact on quality of physical health, the status of mental health might be relatively unaffected. [7], [12], [20], [21] Other studies also suggested that the major contribution of the chronic disease to the negative impact on HRQoL could be the symptoms and disabilities of the disease rather than the disease itself. [9], [12], [14], [21] Besides, Spitzer et al. already proposed that both physical and mental disorders might have different effects on the components of HRQoL. [22] These seemingly controversial results raised the question that, even with similar outcomes, the underlying pathways for these disease to influence the HRQoL might be different, just as that proposed by Mayo et al., who used the Wilson and Cleary model to evaluate the HRQoL in people recovering from stroke. [23] Steca et al. also found a significant relationship of illness severity (predicted by left ventricular function) with health satisfaction and depression in cardiovascular disease patients, but these relationships were fully mediated by illness perception and self-efficacy beliefs (both were indicators of patients’ psychological well-being). [24] Therefore, as mental disorders (such as anxiety and depression) are frequently comorbid with cardiovascular disease and stroke, [25]–[30] whether these two diseases influence the HRQoL directly or indirectly through some other mediators, such as psychological functions, requires further evaluation.

The purpose of this study is trying to determine how psychological factors affect the link between cardiovascular disease and stroke with the HRQoL. Additionally, the impacts of these diseases on the HRQoL will also be evaluated.

## Method

### 1. Participants

This study is a part of the Yilan Study, which is a population-based community health survey conducted by the Community Medicine Research Center at the National Yang-Ming University and the National Yang-Ming University Hospital in Taiwan. Since 2012, with the help of the Community Angels (social volunteers) and the Yang-Ming Crusade (student volunteers of the National Yang-Ming University), all people at least 65 years of age living in the 7 most populated villages of Yilan City, a city located in the northeastern part of Taiwan, have been visited at their homes by well-trained project assistants. In addition to recording the body weight and height, the demographic data, lifestyles and a self-reported medical history, including education level, living status, smoking and drinking habits, hypertenion, diabetes, cardiovascular disease (including coronary heart disease, heart failure and valvular heart disease), stroke, and hyperlipidemia were collected. Then, measurement of anxiety and depression tendency and the HRQoL were also performed. All the questionnaires have been completed by trained project assistants through face to face interview. Those people who did not agree to join the project, could not provide past medical history, had never visited a physician to confirm a medical diagnosis, or could not complete the questionnaires were excluded. All participants provided written informed consent and the Institutional Review Board of the National Yang-Ming University Hospital approved this study (IRB No. 2011A016).

### 2. Measurement of anxiety and depression symptoms

Measurement of anxiety and depression in study participants was performed by use of the Hospital Anxiety and Depression Scale (HADS). The HADS is a short, simple and reliable method for measuring both clinical and subclinical anxiety and depression symptoms in a general clinical setting and general population. [31], [32] The scale is composed of 14 items designed to obtain the participant’s feelings in the past one week, with 7 items rating anxiety and 7 rating depression. Each item is scored from 0 to 3 and the total range of both anxiety and depression subscores are 0–21, with higher scores indicating more anxious and depressive tendency. The Chinese translation of the HADS has been proven valid with the optimal cut-off points of 3 for the anxiety subscore and 6 for the depression subscore [33], [34].

### 3. Measurement of HRQoL

We used the Short Form 12 Health Survey Version 2 (SF-12v2) to evaluate the HRQoL of study participants. Twelve items derived from the Short Form 36 Health Survey Version 2 (SF-36v2) are used to constitute the mental component summary (MCS) and physical component summary (PCS) for measuring the participant’s mental and physical function status in the past four weeks. [35], [36] The SF-36 is one of the most widely used measurements of the HRQoL of people in the world, and the SF-12 has been shown to closely mirror the results. [37], [38] The translation of SF-36 and SF-12 Health Survey from English into Chinese has been carefully developed and proven to be valid. [39]–[41] Besides, because the mean of the norm during standardization was 50, we also divided the results of the MCS and the PCS with the cutoff points of 50 for purposes of additional comparisons. [42] For further validation of the HRQoL, two more questions have been asked: “How do you rate your happiness?” and “How do you rate your health status?” Each item score ranges from 0 to 100. A higher score indicates a greater level of happiness or a healthier life.

### 4. Statistical analysis

The IBM SPSS Statistics version 21 has been used to analyze all data. In the univariate analysis, the independent *t-*test and the one-way analysis of variance (ANOVA) were utilized. The Pearson Correlation coefficient was used to measure the correlations between MCS vs happiness rating, and PCS vs health status rating. Those variables found to be associated with significantly different results of HRQoL by literature review and by univariate analysis were included in the multiple regression analysis. Then, for statiscal efficiency, we choose stepwise multiple regression method to analyze the relationships between HRQoL (MCS & PCS) and major factors of lifestyle and comorbidity in order to find out those variables playing the major roles in the association. The dichotomous values of the HADS, MCS and PCS were used for univariate analysis, and the row scores of them for multivariate and mediation analysis. All reported *p* values are two-tailed and are considered significant if *p*≤0.05.

We used the SPSS macro provided by Preacher and Hayes in the assessment of the direct and indirect effects of cardiovascular disease and stroke affecting the HRQoL. [43] This macro utilizes a bootstrapping strategy to test the validity of the indirect effects and is able to partial out the effects of covariates without the assumption of multivariate normal distribution. [44] In the present study, each test would be resampled 5,000 times to estimate the bias-corrected and accelerated 95% confidence interval (BCa 95% CI). In the multiple mediation model, depression and anxiety were hypothesized to be intervening variables between medical illness and HRQoL. The SPSS macro estimated the direct effect that medical illness confers on HRQoL and provided the estimated indirect effects that were mediated by all intervening variables. All the effects of other factors have also been controlled by this Macro during the analysis (as showing in the figure legends).

## Results

There was a total of 1,285 elderly study participants (≥65 years) with 606 male participants comprising 47.2% of that group. The mean age was 74.6±6.9 years with 56.1% of the participants between 65–75 years of age. The prevalences of the reported medical conditions of these participants were hypertension (51.3%), cardiovascular disease (27.1%), hyperlipidemia (22.4%), diabetes (21.4%), and stroke (7.0%). The mean anxiety subscore, depression subscore, MCS and PCS were 2.5±3.2, 2.7±3.0, 57.5±9.1 and 47.7±10.5, respectively. All the relevant data are shown in Supporting Information S1 and Supporting Information S2.

The MCS and PCS were different in elderly subjects with different lifestyles and comorbidities. Table 1 summarized the results of univariate analysis of HRQoL and showed that male and younger participants with higher education level had higher MCS scores. On the contrary, those who had quit smoking and drinking had lower MCS score. Regarding PCS, people with younger age, normal or slightly higher BMI, higher education level, solitary living status, and status of current drinker obtained a higher measurement of physical health quality. Those people with cardiovascular disease, stroke, and depression had both poorer MCS and PCS measurements. Besides, those participants with anxiety and PCS over 50 typically had lower MCS scores, and those with diabetes, hypertension, and MCS over 50 showed poorer PCS. Significant positive correlations between MCS vs happiness, and PCS vs health status were also noted (MCS/happiness rating: *r* = 0.336, *p*<0.001, PCS/health status rating: *r* = 0.365, *p*<0.001).

**Table 1:** Univariate analysis of health-related quality of life among the elderly in Yilan from 2011–2012.

	Mental component score					Physical component score			
	n	mean	(SD)		P	n	mean	(SD)	P
Sex					**0.010**				0.644
Male	593	58.1	8.5			593	47.9	(11.1)	
Female	654	56.8	9.6			654	47.6	(10.0)	
Age					**0.001**				**<0.001**
65–<70 years	354	58.8	(7.7)			354	49.6	(8.9)	
70–<75 years	349	57.6	(9.3)			349	49.0	(8.8)	
75–<80 years	255	57.3	(8.5)			255	46.7	(11.6)	
80 years above	289	55.8	(10.6)			289	44.7	(12.5)	
BMI					0.209				**0.005**
<18.5	56	56.6	(8.3)			56	46.2	(12.8)	
18.5–<24	504	57.3	(8.6)			504	48.8	(9.8)	
24–<27	374	58.0	(9.0)			374	49.1	(9.3)	
≥27	267	58.5	(8.3)			267	46.7	(10.2)	
Education					**<0.001**				**<0.001**
Illiterate	204	55.8	(9.8)			204	45.3	(11.0)	
Primary school	461	56.8	(9.8)			461	47.3	(11.2)	
Secondary school	363	58.3	(8.2)			363	48.9	(9.6)	
College and above	207	59.3	(7.4)			207	49.1	(9.5)	
Living status					0.076				**0.010**
Solitary	127	56.1	(9.3)			127	49.8	(9.6)	
Not solitary	1,120	57.6	(9.1)			1,120	47.5	(10.6)	
Smoking					**0.010**				0.094
Never	969	57.5	(9.0)			969	48.0	(10.3)	
Current smoker	123	59.2	(7.9)			123	47.3	(10.4)	
Quitted	155	55.8	(10.2)			155	46.1	(12.2)	
Drinking					0.065				**0.001**
Never	1,009	57.3	(9.2)			1,009	47.3	(10.6)	
Current drinker	175	58.8	(7.3)			175	50.5	(9.0)	
Quitted	63	56.3	(10.8)			63	46.8	(12.9)	
Hyperlipidemia					0.541				0.310
No	964	57.5	(9.1)			964	47.9	(10.6)	
Yes	278	57.1	(9.2)			278	47.2	(10.1)	
Diabetes					0.514				**0.003**
No	979	57.5	(8.9)			979	48.2	(10.3)	
Yes	266	57.1	(9.7)			266	45.9	(11.2)	
Hypertension					0.655				**0.015**
No	602	57.6	(8.9)			602	48.5	(10.2)	
Yes	644	57.3	(9.3)			644	47.0	(10.8)	
Cardiovascular disease					**<0.001**				**0.001**
No	902	58.1	(8.7)			902	48.4	(10.1)	
Yes	336	55.9	(9.9)			336	46.0	(11.4)	
Stroke					**0.016**				**<0.001**
No	1,163	57.7	(8.7)			1,163	48.3	(10.0)	
Yes	79	53.9	(13.3)			79	39.4	(14.1)	
Hospital Anxiety and Depression Scale									
Anxiety subscore					**<0.001**				0.570
<3	781	59.8		(6.8)		781	47.9	(10.2)	
≥3	460	53.7		(10.8)		460	47.6	(11.0)	
Depression subscore					**<0.001**				**<0.001**
<6	1,052	58.7		(8.0)		1,052	48.9	(9.8)	
≥6	189	51.1		(11.4)		189	41.4	(11.9)	
Short form 12 Health Survey									
Physical component score					**0.001**				
<50	584	58.4		(9.7)					
≥50	658	56.6		(8.5)					
Mental component score									**0.043**
<50						226	49.3	(13.5)	
≥50						1,021	47.4	(9.8)	

Table 2 showed the results of multiple regression analysis of HRQoL with different lifestyles and comorbidities. After controlling for possible confounding effects, anxiety, depression, age, education level, cardiovascular disease and stroke were shown to be significantly correlated with both MCS and PCS. We also found that BMI and quit smoking status were correlated with MCS and diabetes with PCS. Besides, the MCS and PCS were negatively correlated with each other. The R square of the final selected model was 0.390 in MCS and 0.323 in PCS, as compared to 0.395 of the full model in MCS and 0.329 in PCS.

**Table 2:** Multiple regression analysis for factors associated with health-related quality of life among the elderly in Yilan from 2011–2012.

	Mental component score				Physical component score		
	Beta	*P*	Cumulative R^2^		Beta	*P*	Cumulative R^2^
**Final stepwise model**				**Final stepwise model**			
(Constant)	86.877	<0.001		(Constant)	100.546	<0.001	
Anxiety	−0.832	**<0.001**	0.173	Depression	−1.422	**<0.001**	0.103
Physical component score	−0.366	**<0.001**	0.244	Mental component score	−0.536	**<0.001**	0.250
Depression	−1.068	**<0.001**	0.355	Age	−0.231	**<0.001**	0.277
Age	−0.144	**<0.001**	0.373	Stroke	−5.489	**<0.001**	0.292
Education	0.748	**0.001**	0.378	Cardiovascular disease	−2.100	**<0.001**	0.303
Cardiovascular disease	−1.567	**0.001**	0.383	Anxiety	−0.297	**<0.001**	0.311
Stroke	−2.848	**0.002**	0.388	Diabetes	−2.007	**0.001**	0.319
Body mass index	0.123	**0.029**	0.390	Education	0.710	**0.006**	0.323
Forced-enter full model			0.395	Forced-enter full model			0.329
Controlling Variables: sex, living status, smoking∼current,smoking∼quit, drinking∼current, drinking∼quit, diabetes,hypertension, and hyperlipidemia.				Controlling Variables: sex, body mass index, living status,smoking∼current, smoking∼quit, drinking∼current, drinking∼quit,hypertension, and hyperlipidemia.			

**Figure 1:**
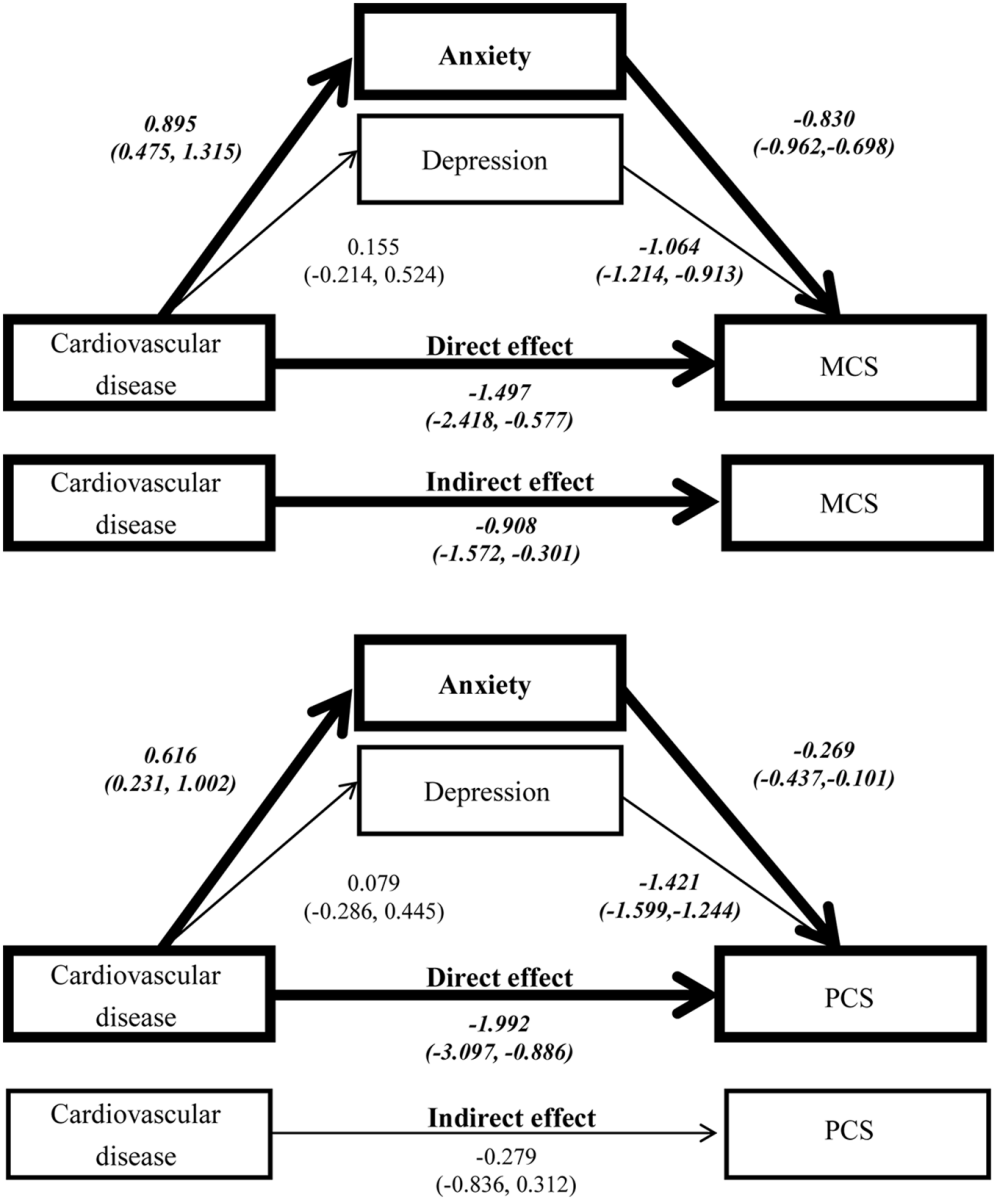
The estimation of the direct and indirect effect of cardiovascular disease on MCS & PCS (Bootstrap resamples: 5000). Footnote: Controlling covariates: Age, sex, body mass index, education, living status, smoking, drinking, diabetes, hypertension, hyperlipidemia, stroke, and PCS (or MCS). MCS: mental component score, PCS: physical component score, (): bias-corrected and accelerated 95% confidence interval. Bolded lines indicate significant direct and indirect effects.

**Figure 2:**
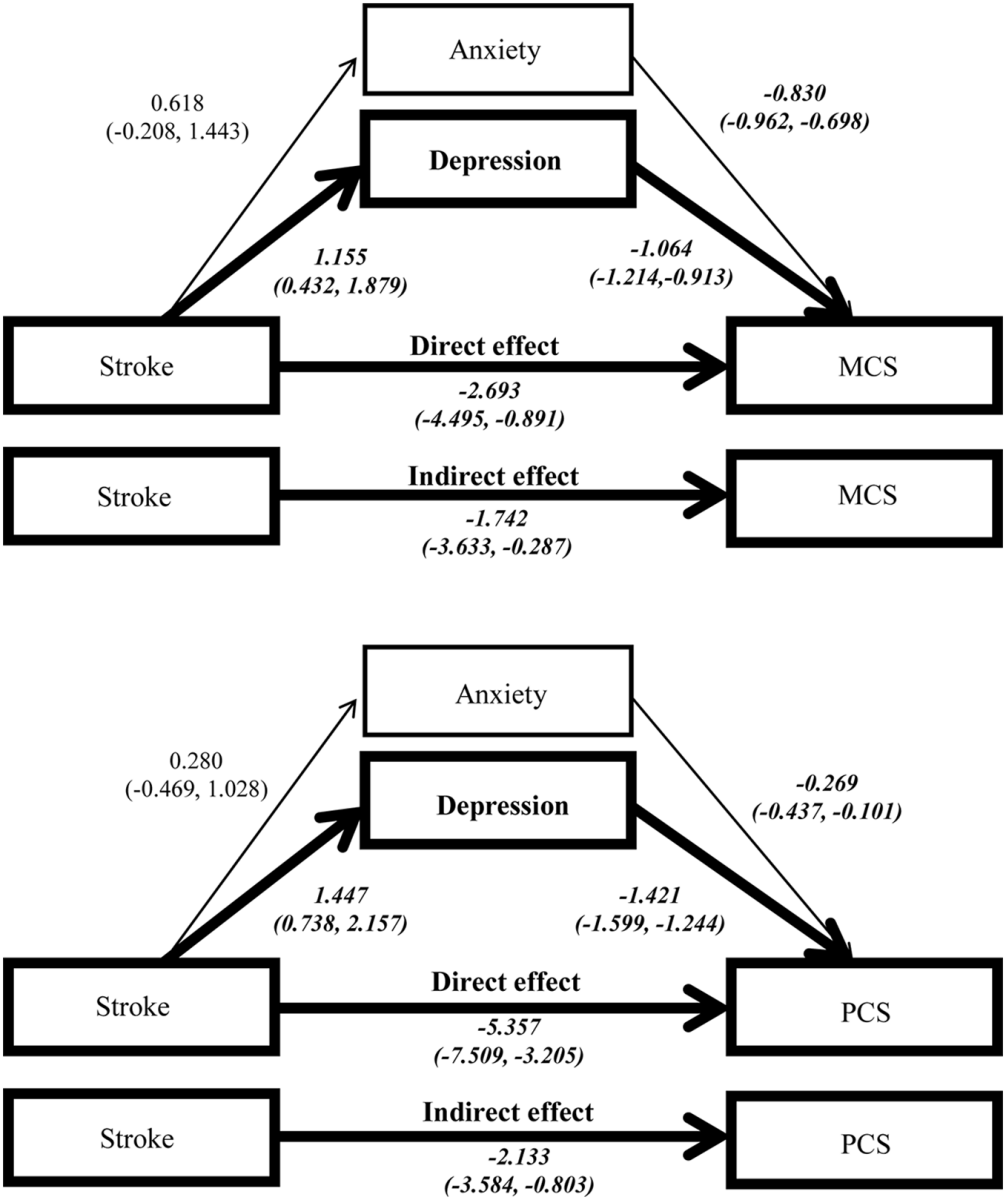
The estimation of the direct and indirect effect of stroke on MCS & PCS (Bootstrap resamples: 5000). Footnote: Controlling covariates: Age, sex, body mass index, education, living status, smoking, drinking, diabetes, hypertension, cardiovascular disease, hyperlipidemia, and PCS (or MCS). MCS: mental component score, PCS: physical component score, (): bias-corrected and accelerated 95% confidence interval. Bolded lines indicate significant direct and indirect effects.

Another interesting finding was that anxiety entered the model earlier than depression in the regression analysis of MCS, but vice versa in that of PCS. Besides, cardiovascular disease also entered the model earlier than the stroke in MCS analysis, but with reversed sequence in PCS analysis. The different sequences meant various impacts of these variables on the MCS and PCS. Therefore, it would be reasonable to assume that psychological factors might influence the link of cardiovascular disease and stroke with HRQoL by different pathways. Figure 1 and 2 shows the results of the estimation of the pathways that cardiovascular disease and stroke affect HRQoL. These figures suggested that in addition to the direct effect, cardiovascular disease exerted its impact on the HRQoL (both MCS & PCS) indirectly through anxiety. On the contrary, stroke affected the MCS and PCS both directly and indirectly through depression.

## Discussion

In this study, we found that cardiovascular disease and stroke are both negatively associated with health related quality of life through different pathways. The cardiovascular disease influences the MCS and PCS both directly and indirectly through anxiety. On the other hand, with a direct effect on both kinds of HRQoL, stroke also has an impact on MCS and PCS mediated by depression indirectly. These results provide an evidence to clarify the possible underlying mechanisms between HRQoL and cardiovascular disease and stroke. Depression and anxiety are common comorbidities in patients with cardiovascular disease and stroke. [25]–[30], [45]–[50]. There are several assumptions for such high prvalence. First, both depression and anxiety have been proposed to be the risk factors or preictors of cardiovascular disease and stroke. [25], [27], [29], [45] Second, cardiovascular disease and stroke may also precipitate the development of depression and anxiety through biological and behavioral mechanisms. From biological aspects, Hare et al. have summaried the possible mechanisms of post-cardiovascular disease depression, which included alterations in the autonomic nervous system, platelet receptors and function, coagulopathic factors (such as plasminogen activator inhibitor-1 and fibrinogen), pro-inflammatory cytokines, endothelial function, neurohormonal factors, and genetic linkages. [29] Feng C. also reviewed the possible neurobiological pathogenesis for post-stroke depression, which included neuroanatomical factors (lesion location, infarction size, vascular depression hypothesis), neuronal biochemical factors (neurotransmitter, immune dysfunction, hypothalamic-pituitary-adrenal axis activation), and neurogenesis hypothesis. [51] Hellmann-Regen J at al. even proposed that stroke might share many pathophysiological features and risk factors with cardiovascular disease in the association with depression. [45] Besides, though there were relatively less researches about whether cardiovascular disease and stroke might precipitate the development of anxiety, Hare and Knutson have still suggested that both diseases might lead the development of anxiety through at least partially similar pathogenesis as those of the development of depression. [29], [52] From behavior aspects, Hare at al. have summarized that cardiovascular disease might be associated with perceived loss (such as health, functional capacity, employmernt, and independence) and social isolation, which might lead to depression and anxiety. [29] Afanasiev et al. also suggested that physical disability, stroke severity, and cognitive decline were so far the main predictors of depressive symptoms. [53] Besides, there were also studies suggested that the anxious symptoms induced by fears (such as the recurrent attacks of disease, falling and returning to work) after disease attacks might influence the daily life. [27] Both depression an anxiety have been proposed to be negatively associated with HRQoL. [17], [22], [27], [29], [30], [46], [48], [50], [54]–[56] Our study results show similar results, however, that the mediators of the effects of both diseases on the HRQoL are different. We believe the neuroanatomic factors (such as the lesion location and infarction size) of stroke and neurogenesis post stroke, both involving the neurological pathways mediating the emotional responses and being different from those possible mechanisms of post-cardiovascular depression, might be two of the possible reasons for such difference. [51] However, more further detailed investigations to valide the porposal are necessary. Besides, we also believe that the reversibility of the symptoms of these two diseases might also play an important role. For example, most of the symptoms (such as dyspnea, angina, etc.) of the cardiovascular disease are reversible, therefore making those patients prone to worry about when and how to return the work and thus precipitated them to anxiety. On the other hand, those stroke patients usually got irreversible physical disability, which has been one of the predictors of depression. Our results also support Mayo et al.’s hypothesis that with similar outcomes of HRQoL across various health conditions, the pathways to these outcomes might be different. [23] Besides, the various pathways may also have important clinical implications. For example, in those people with cardiovascular disease, management of disease progression alone may not be enough. Adequate education to promote good illness perception with resultant positive self-efficacy belief, as proposed by Steca et al., should also be included to relieve the possible anxiety of these people. [24] On the other hand, for those people with stroke, because of the high prevalence of depression after stroke, [30] the goal of treatment should focus on both functional recovery and psychological support, as proposed by Mayo et al., who has suggested that interventions should not only focus on functional recovery during the first 3 months post-stroke. [23] However, this assumption of different pathways still needs further evaluation and validation.

Though both anxiety and depression are negatively associatd with HRQoL, the early entrance into the regression model with higher R square suggests that anxiety might have a greater correlation with MCS than depression, and depression is correlated with PCS more closely than that of anxiety. The findings suggest that various emotional responses to these diseases might influence the HRQoL components differently. The explanation of this result might be partially attributed to the various underlying mechanisms precipitating the development of these emotional responses. These different patterns also give support to Spitzer’s presumption. [22] Besides, the results further suggest that responsible management of mental functions in patients with cardiovascular disease and stroke may become one of the crucial issues.

After controlling for lifestyles, mental functions, and other medical conditions, our results show that cardiovascular disease and stroke are still negatively correlated with HRQoL, which are compatible with those results from the EUROASPIRE III, IQOLA, etc. [6], [12], [20] Previous studies have suggested that diseases which are more symptomatic and disabling have increased negative impacts on the HRQoL. [12], [21] In our study, we believe those patients with cardiovascular disease and stroke would suffer from morefunctional deteriorations, such as angina, dyspnea on exertion, lower leg edema, and paralysis, than those of patients with other kind of diseases. These functional deteriorations, without the mediation of the negative emotional responses, would also directly decrease the HRQoL. However, the underlying mechanisms and whether other kinds of chronic disease might have such similar effects still need further evaluation.

Our study results also show that both MCS and PCS are significantly but negatively associated with each other. Taft et al. have suggested that because of the construction of the summaries, there might be reciprocal effects of subscale scores on PCS and MCS, especially in those cases with extremely unbalanced profiles. [57], [58] Thus, high MCS may represent either good mental health or poor physical status, and vice versa. Therefore, clinical interpretation of MCS and PCS scores should be in conjunction with the assessment of patient well-being and functioning. [57] The positive correlations between MCS/happiness and PCS/health status in our study suggest that the results of SF-12V2 have faithfully reflected the real status of the participants, and the negative association between MCS and PCS might have been resulted from their positive mental satisfaction.

There are still some limitations in this study. First, although we aggressively attempted to visit all the elderly adults dwelling in this area, nearly 30% of the registered inhabitants refused to join this study. Though the age and sex distribution were similar between the participants and all registered inhabitants, those who did not agree to join the study could be those suffering from more physical or mental disabilities. This could lead to an underestimate of the correlation between diseases and HRQoL. Second, some of the participants might have diseases but they have not previously requested any medical help, so the prevalence of diseases might also be underestimated. Third, as proposed by Tseng et al., there might be some cultural influences upon illness attribution and perception in the measurement of HRQoL. Therefore our results should be interpreted taking these concerns into consideration. [40] Finally, though there are some overlapping of the pathophysiological features between cardiovascular disease and stroke, there are still some differences among these diseases. [29], [45] Therefore further analysis of the interrelationship between HRQoL with each individual disease are indicated. However, the results of the present study still suggest that different diseases might have different effects on the HRQoL, and even with different pathways. These findings also provide the basis for further analysis.

In conclusion, after controlling for possible confounders, our study results have suggested that cardiovascular disease and stroke have negative impacts on both the mental and physical part of HRQoL through different pathways. Separate and apart from the direct effect, cardiovascular disease affects the HRQoL indirectly with the mediation of anxiety while stroke with depression. These findings support the proposition that different combinations of physical and psychological support in the managements of these diseases are necessary.

## Supporting Information

Supporting Information S1 Dataset.
(XLSX)

Supporting Information S2 Meta file.
(XLSX)
